# *spa* Typing of Methicillin-Resistant Staphylococcus aureus Based on Whole-Genome Sequencing: the Impact of the Assembler

**DOI:** 10.1128/spectrum.02189-22

**Published:** 2022-11-09

**Authors:** Sarah Mollerup, Peder Worning, Andreas Petersen, Mette Damkjær Bartels

**Affiliations:** a Department of Clinical Microbiology, Copenhagen University Hospital - Amager and Hvidovre, Copenhagen, Denmark; b Department of Bacteria, Parasites & Fungi, Statens Serum Institut, Copenhagen, Denmark; c Department of Clinical Medicine, University of Copenhagen, Copenhagen, Denmark; University of Calgary

**Keywords:** MRSA, WGS, Sanger, *spa* typing, assembly, SKESA, SPAdes

## Abstract

Sequencing of the *spa* gene of methicillin-resistant Staphylococcus aureus (MRSA) is used for assigning *spa* types to e.g., detect transmission and control outbreaks. Traditionally, *spa* typing is performed by Sanger sequencing but has in recent years been replaced by whole-genome sequencing (WGS) in some laboratories. *Spa* typing by WGS involves *de novo* assembly of millions of short sequencing reads into larger contiguous sequences, from which the *spa* type is then determined. The choice of assembly program therefore potentially impacts the *spa* typing result. In this study, WGS of 1,754 MRSA isolates was followed by *de novo* assembly using the assembly programs SPAdes (with two different sets of parameters) and SKESA. The *spa* types were assigned and compared to the *spa* types obtained by Sanger sequencing, regarding the latter as the correct *spa* types. SPAdes with the two different settings resulted in assembly of the correct *spa* type for 84.8% and 97.6% of the isolates, respectively, while SKESA assembled the correct *spa* type in 98.6% of cases. The misassembled *spa* types were generally two *spa* repeats shorter than the correct *spa* type and mainly included *spa* types with repetition of the same repeats. WGS-based *spa* typing is thus very accurate compared to Sanger sequencing, when the best assembly program for this purpose is used.

**IMPORTANCE** *spa* typing of methicillin-resistant Staphylococcus aureus (MRSA) is widely used by clinicians, infection control workers, and researchers both in local outbreak investigations and as an easy way to communicate and compare MRSA types between laboratories and countries. Traditionally, *spa* types are determined by Sanger sequencing, but in recent years a whole-genome sequencing (WGS)-based approach has become increasingly used. In this study, we compared *spa* typing by WGS using different methods for assembling the genome from short sequencing reads and compared to Sanger sequencing as the gold standard. We find substantial differences in correct assembly of *spa* types between the assembly methods. Our findings are therefore important for the quality of WGS based *spa* typing data being exchanged by clinical microbiology laboratories.

## INTRODUCTION

Typing of methicillin-resistant Staphylococcus aureus (MRSA) is used to detect transmission of MRSA and to describe the epidemiology and evolution of MRSA clones. One of the most used typing methods for MRSA is *spa* typing, which is based on sequencing of the repeat region of the *spa* gene (1, 2). The implementation of the Ridom StaphTyper software in 2003 ensured a uniform and updated terminology (3) and allowed clinicians, infection control teams, and researchers to share and compare their data. Traditionally, *spa* typing has been performed by Sanger sequencing. However, in recent years, many clinical microbiology laboratories have implemented whole-genome sequencing (WGS) as a routine method, and *spa* types are extracted from the assemblies.

At Hvidovre Hospital, we performed Sanger sequencing of the *spa* gene from 2003 to 2012, and since 2013, WGS has been performed routinely on first time MRSA isolates from all patients. We have previously shown that *spa* types based on WGS data had a 97% concordance with *spa* types found by Sanger sequencing in a diverse collection of 699 isolates from Copenhagen (4). In that study, we used Velvet as the assembler, but over the years we have used several different assemblers and have found that *spa* type results can differ depending on the assembler used. Since the end of 2015 until December 2020, we have *de novo* assembled whole-genome sequenced MRSA isolates using SPAdes (5), and in December 2020 we switched to SKESA (6). In the present study, we compare the *spa* typing results based on SPAdes run with two different sets of parameters and SKESA to Sanger sequencing, the latter regarded as the gold standard.

## RESULTS

### Overall performance of the methods compared.

Comparison of *spa* typing results was performed for 1,754 isolates (see Table S1 in the supplemental material). The collection of isolates was very diverse and included 263 different *spa* types as determined by Sanger sequencing. For 1,478 (84.3%) of the included isolates, all four methods resulted in the same *spa* type. These isolates represented 225 different *spa* types. Of these, 172 *spa* types were always correctly assembled (78 when disregarding *spa* types only appearing once) (Table S2).

**FIG 1:**
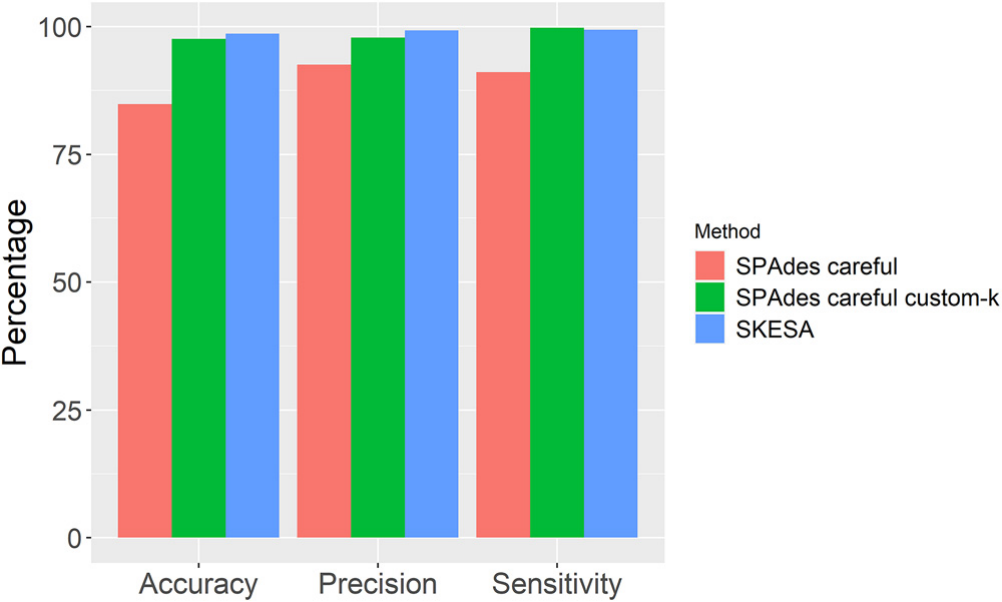
Performance metrics for the three methods. Accuracy, precision, and sensitivity of *spa* type determination are shown for SPAdes careful, SPAdes careful custom-k, and SKESA.

Differing *spa* types (including no *spa* type) were found for the remaining 276 isolates (Table S3). Of the applied methods, SKESA performed the best when comparing to Sanger sequencing, with the wrong or no *spa* type found for 13 and 11 isolates, respectively (98.6% accuracy) (Fig. 1). SPAdes careful custom-k found the wrong or no *spa* type for 38 and 4 isolates, respectively (97.6% accuracy), while the wrong or no *spa* type was found for 120 and 146 isolates, respectively, (84.8% accuracy) for SPAdes careful. A *spa* type could not be assigned for two isolates (0.001%) by Sanger sequencing. For one of these isolates, all three assembly methods identified the same *spa* type, while for the other isolate, SPAdes careful custom-k and SKESA found the *spa* type t223, while SPAdes careful found the *spa* type t3243. Overall, SKESA and SPAdes careful custom-k performed the best concerning both accuracy, precision, and sensitivity (Fig. 1).

### Most frequently misassembled *spa* types.

Of the 263 represented *spa* types, 91 were misassembled or not assigned more than once (Table S3) and of these, 24 *spa* types were misassembled more than once by the same method (Table 1). The *spa* type misassembled the most times by SPAdes careful was t034. Eighty-five isolates were t034 by Sanger sequencing, but only one of these was found to be t034 by SPAdes careful (1.1%). The *spa* types found by SPAdes careful for the remaining t034 isolates were t011 (51 isolates), t4677 (16 isolates), t1170 (nine isolates), t7880 (one isolate) and seven isolates with no *spa* typing result. In addition, all eight t032 were either misassembled by SPAdes careful as t379 (7 isolates) or had no *spa* typing result (1 isolate), and t15676 was found to be t1358 in three of three cases.

**TABLE 1:** *spa* types wrong or missing more than once for the same method^*a*^

		SPAdes careful		SPAdes careful custom-k		SKESA	
Sanger *spa* type	Isolates (*n*)	Type wrong	Type missing	Type wrong	Type missing	Type wrong	Type missing
t002	77		9 (12%)			2 (3%)	
t005	70		7 (10%)				
t008	108		11 (10%)				
t019	33		6 (18%)				
t021	41		2 (5%)				
t032	8	7 (88%)					
t034	85	77 (91%)	7 (8%)	18 (21%)			
t148	14		2 (14%)				
t216	11		2 (18%)				
t223	84	3 (4%)	13 (15%)				
t304	233		11 (5%)				
t309	16		3 (19%)				2 (13%)
t316	6			2 (33%)		2 (33%)	
t324	2		2 (100%)				
t355	6		2 (33%)				
t359	13		2 (15%)				
t521	3	2 (67%)					
t852	7	2 (29%)					
t2582	2		2 (100%)	2 (100%)			
t3841	17		4 (24%)				
t4652	4	2 (50%)	2 (50%)				
t13748	13		2 (15%)				
t15676	3	3 (100%)					
t19381	4		3 (75%)		2 (50%)		
Isolates (*n*)		96	92	22	2	4	2
*spa* types (*n*)		7	19	3	1	2	1

a The number of included isolates with a given *spa* type is shown, as well the number and percentage of times the *spa* type was found wrong or missing (Type wrong/missing) when comparing to the *spa* type found by Sanger. Only *spa* types wrong or missing more than once for a given assembler are included in the table.

For SPAdes careful custom-k, t034 was also the *spa* type misassembled the most times, with 18 of 85 isolates (21%) having the wrong *spa* type assigned, in 17 cases as t011, and in one case as t1170. For SKESA, the most frequently misassembled *spa* type was t316, being misassembled in two of six cases (33%).

### Characteristics of misassembled *spa* types.

To investigate potential reasons for misassembly of *spa* types, we compared the repeat composition of *spa* types with high frequency of misassembly (Table 2). For many of the misassemblies, the disagreements were due to repetition of the same sequence of repeats not being assembled correctly, i.e., 02-25-02-25 in t034 assembled as 02-25 or 31-29-17-31-29-17 in t032 assembled as 31-29-17. We also analyzed the length of the misassembled *spa* types and found that in general, the misassembled *spa* types were shorter than the *spa* types found by Sanger sequencing (Table 3). For all included isolates, the median number of repeats in the Sanger *spa* types was nine (range 2 to 16), while the median missassembled *spa* types were 2 repeats shorter. The median number of repeats for the *spa* types that could not be assembled was higher than the overall median number of repeats. No *spa* types shorter than 5 repeats were misassembled by any method, and no *spa* types of 15 or 16 repeats were correctly assembled by all methods.

**TABLE 2:** Comparison of *spa* repeats for *spa* types often misassembled by SPAdes careful

Method	*spa* type	*spa* repeats
Sanger	t034	08-16-02-25-02-25-34-24-25
SPAdes careful	t011	08-16-02-25-34-24-25
Sanger	t034	08-16-02-25-02-25-34-24-25
SPAdes careful	t4677	08-16-02-25-02-24-25
Sanger	t034	08-16-02-25-02-25-34-24–25
SPAdes careful	t1170	08-16-02-25-02-25-25
Sanger	t032	26-23-23-13-23-31-29-17-31-29-17-25-17-25-16-28
SPAdes careful	t379	26-23-23-13-23-31-29-17-25-17-25-16-28
Sanger	t15676	26-23-13-23-31-29-17-25-17-25-25-17-25–28
SPAdes careful	t1358	26-23-13-23-31-29-17-25-17-25–28

**TABLE 3:** Median number of repeats in the wrong or missing *spa* types^*a*^

	Misassembled *spa* types				No *spa* type	
Method	Isolates (*n*)	*spa* type shorter than Sanger *spa* type (*n*)	No. of repeats, Sanger *spa* type, median (range)	No. of repeats, assembled *spa* type, median (range)	Isolates (*n*)	No. of repeats, Sanger *spa* type, median (range)
SPAdes careful	119	115	9 (5–16)	7 (3–14)	146	10 (6–16)
SPAdes careful custom-k	38	33	9 (5–14)	7 (3–14)	4	13.5 (12–15)
SKESA	13	8	10 (5–12)	8 (3–11)	11	11 (6–15)

a The number of isolates with wrong or missing *spa* types and the median number of *spa* repeats as well as the range for both assembled *spa* types and Sanger *spa* types are shown.

### Other discordances.

Besides the above-mentioned *spa* types that were more frequently misassembled, the remaining misassignments represented sporadic misassemblies. For 25 isolates, more than one method found a different *spa* type than the one determined by Sanger sequencing (excluding t011/t034 misassemblies; Table S3). For some of these, two or all three assembly methods found the same *spa* type (differing from Sanger). In most of these cases, the incorrect *spa* types found were composed of many of the same repeats as the *spa* types found by Sanger sequencing. For four isolates (M8252, M7568, M8788, and M9094), however, the *spa* types found by and agreed upon by all three methods was unrelated to the one found by Sanger sequencing. These differences could either reflect sample mixup or be due to simultaneous carriage of two different MRSA types.

## DISCUSSION

From the end of 2015 to December 2020, our lab was assembling WGS isolates using SPAdes. As we have previously experienced challenges with assembling specific *spa* types such as t034, all MRSA genomes were routinely assembled with SPAdes careful and SPAdes careful custom-k concurrently, and in case of disagreeing results, additional assemblers (MIRA and Velvet) (7, 8) were run, and the *spa* type was chosen based on consensus between at least two assemblers when possible. After switching the assembler to SKESA, we hereby present a systematic comparison of *spa* types obtained using SKESA, SPAdes careful, and SPAdes careful custom-k.

Our study shows that *spa* typing based on WGS assemblies are highly congruent with Sanger sequencing, when choosing the best assembler for this purpose. We found a 98.6% agreement between *spa* types found by Sanger sequencing and WGS assemblies when using SKESA and 97.6% using SPAdes careful custom-k. However, SPAdes careful only had an 84.8% agreement with Sanger sequencing. For De Bruijn graph-based assemblers, such as SPAdes and SKESA, the parameter k determines the size of the k-mers into which the sequencing reads are cut up during assembly. Having repeats longer than k nucleotides can therefore tangle the assembly graph and result in break-up of contigs (9). According to the SPAdes manual, the default set of k-mers used for Illumina 2 × 150 bp reads are 21,33,55,77. Using longer k-mers when running SPAdes as for SPAdes careful custom-k (33,55,77,99,121), will therefore result in better assembly of long repetitive sequences, such as those making up the *spa* type. In contrast to SPAdes, SKESA generates k-mers that are longer than the reads (and up to the library insert size) from mini-assemblies of a subset of reads. Using k-mers longer than the read length will result in more accurate assembly of regions with repetitive sequences shorter than the library insert size but longer than the read length (6), explaining the higher success rate of SKESA in correctly assembling the *spa* repeats. Based on our results, SPAdes careful cannot be recommended as a stand-alone assembler for *spa* typing, or other applications where assembly of repetitive regions are important, without optimization of the k-mers used for assembly.

The disagreements between *spa* types obtained by Sanger sequencing and WGS in this study was mainly caused by *spa* types with repetition of the same repeats. This is consistent with the findings in our previous study (4) and it is a well-known problem that repetition of the same repeats located on different sequencing reads can lead to misassemblies (10). Although t034, having a repetition of two repeats (02-25-02-25), was generally difficult to assemble, especially for SPAdes careful, the similarly abundant *spa* type t223, also having a repetition of two repeats (17-25-17-25), was assembled correctly in 82% of cases by SPAdes careful. The *spa* types t034 and t223 consist of nine and 11 repeats, respectively, so in both cases the *spa* sequence will be on at least two sequencing reads. For t034, the repeated repeats occur in the middle of the *spa* type (08-16-02-25-02-25-34-24-25), while for t223, the repeated repeats occur toward the end of the *spa* type (26-23-13-23-05-17-25-17-25-16-28). This could imply, that the position of the repeated repeats in the *spa* type has impact on how easy or difficult a *spa* type is to assemble.

A standardized approach to validation of WGS-based typing and analysis of microorganisms is not yet established. However, approaches have been made to develop such guidelines and implement quality control metrics (11, 12), some of which were also included in our evaluation. We did not attempt to validate the *de novo* assemblers used in our study as such, merely their ability to correctly assemble *spa* types.

WGS-based core genome multilocus sequence typing (cgMLST) types or single nucleotide polymorphism analyses are much more discriminative than *spa* typing both in outbreak situations and in evolutionary studies, so one could question if *spa* types are still important to report. In our opinion, as clinicians, infection control teams, and researchers have used *spa* typing for many years it is an easy way to communicate in outbreak situations and compare MRSA types between laboratories, hospitals, and countries. For several years we have included the *spa* type in the final report for a sample. In our experience, the *spa* types are easy to remember for the clinicians and makes them more aware of a possible transmission event. However, it is important to be aware that isolates having the same *spa* type can be unrelated and that different *spa* types can be closely related as just one base-pair change in the *spa* gene will change the repeat number and consequently also the *spa* type (13–15).

In conclusion, our study confirms that *spa* typing based on WGS data are very accurate compared to Sanger sequencing, but that the assembler used impacts the agreement between the two methods. In our study, the assembler SKESA performed the best and we therefore recommend SKESA as the assembler of MRSA genomes when *spa* typing is a desired outcome.

## MATERIALS AND METHODS

### Isolates.

All isolates were from the Department of Clinical Microbiology at Hvidovre Hospital, Copenhagen, Denmark. S. aureus isolates suspected to be MRSA based on EUCAST cefoxitin disk diffusion breakpoints were confirmed to be MRSA in an in-house PCR with targets for the genes *nuc*, *fem*A, *mec*A, and *mec*C (primer sequences can be found in Table S4). We included 1,754 consecutive MRSA isolates sequenced between 10 January 2019 and 27 November 2020. All MRSA isolates from both carriage and disease were sent to Statens Serum Institut (SSI), Copenhagen, Denmark, for national surveillance.

Since 2013, the first MRSA isolate of all MRSA-positive patients in the uptake area of Amager and Hvidovre Hospital in the Capital region of Denmark, has been whole-genome sequenced. Consecutive isolates from the same patients are only sequenced if more than a year has passed since the last isolate was sequenced or if the resistance pattern has changed.

### Sanger sequencing.

Sanger sequencing of the *spa* gene is routinely performed at SSI for national surveillance. The isolates were *spa*-typed using a previously described protocol (16) (primer sequences can be found in Table S4). Sequencing of the *spa* amplicons were either done at SSI or sent to Genewiz, Leipzig, Germany. Annotation to *spa* type was done using BioNumerics 8.1 (bioMérieux, Sint-Martens-Latem, Belgium).

### Whole-genome sequencing.

DNA was extracted using the DNeasy blood and tissue kit (Qiagen) from a single colony subcultured in serum broth (SSI Diagnostica, Denmark) and incubated for 24 h. Sequencing libraries were prepared using Nextera XT DNA sample preparation kit (Illumina). The genomes were sequenced with 2 × 150 bp paired-end reads on an Illumina Miseq or NextSeq.

### Bioinformatic analysis.

*De novo* assembly: Raw read files were assembled with SPAdes (5) v. 3.11.1 using either the parameter –careful (SPAdes careful) or the parameters –careful -k 33,55,77,99,121 (SPAdes careful custom-k). The latter combination of k-mers were chosen based on tests of different combinations of k-mers previously run by our lab. Contigs shorter than 500 bp were filtered from the assemblies. For SKESA assembly (6), raw reads were trimmed using bbduk (https://sourceforge.net/projects/bbmap/) with the parameters ktrim=r, k = 23, mink = 11, hdist = 1, tbo, qtrim=r, and minlength = 30. Assembly was performed using SKESA v. 1.2 with default settings except inclusion of the parameter –allow_snps.

Only assemblies with a genome size in the range 2.6 to 3 Mb, a minimum depth of coverage of 30, and *N*_50_ of minimum 10,000 were kept for further analysis and included in the study. The presence of *mecA* was confirmed by an in-house script, and *spa* types were assigned using an in-house script compared to known *spa* types downloaded from the Ridom SpaServer (https://spa.ridom.de/spatypes.shtml). Accuracy, precision, and sensitivity were calculated according to suggestions made by Bogaerts et al. (11) for all three methods:

Accuracy = 100% × (TP + TN)/(TN + FN + TP + FP)

Precision = 100% × TP/(TP + FP)

Sensitivity = 100% × TP/(TP + FN)

where TP indicates true positives; TN, true negatives; FP, false positives; and FN, false negatives. The specificity was not evaluated as this, per definition, requires the existence of true negatives (specificity = 100% × TN/[TN + FP]), which was not found in the present study.

### Data availability.

Raw sequencing reads are deposited in NCBI Sequence Read Archive under BioProject no. PRJNA839593.
